# Transcriptomic Mechanisms Underlying Dietary Fish Oil, Phospholipid, and Vitamin E Supplementation in Promoting Ovarian Development in *Leptobotia elongata*

**DOI:** 10.3390/ani16111604

**Published:** 2026-05-25

**Authors:** Yuxin Jiang, Yihui Mei, Lin Luo, Wenqi Chang, Jian Gao, Min Guan, Xiaojuan Cao

**Affiliations:** 1 College of Fisheries, Engineering Research Center of Green Development for Conventional Aquatic Biological Industry in the Yangtze River Economic Belt, Ministry of Education, Huazhong Agricultural University, Wuhan 430070, China; 18744284955@163.com (Y.J.); myh@webmail.hzau.edu.cn (Y.M.); 16604130503@163.com (W.C.); gaojian@mail.hzau.edu.cn (J.G.); 2 Hubei Key Laboratory of Three Gorges Project for Conservation of Fishes, Yichang 443100, China; luolin20210618@163.com; 3 Chinese Sturgeon Research Institute, China Three Gorges Corporation, Yichang 443100, China

**Keywords:** *Leptobotia elongata*, lipid nutrition, ovarian development transcriptomics, ribosome biogenesis, oxidative stress

## Abstract

*Leptobotia elongata*, a rare freshwater fish endemic to China, frequently exhibits retarded ovarian development under artificial captivity, severely constraining large-scale reproduction. This study evaluated the effects of combined dietary supplementation with fish oil (FO), soybean phospholipids (PLs), and vitamin E (VE) on ovarian development through a 6-month feeding trial. Hematoxylin and eosin staining revealed that oocytes in the mixed lipid group (MIX: FO + PL + VE) advanced to developmental stage III, whereas oocytes in the control group (CON) were predominantly arrested at stages I–II. Transcriptomic analysis identified 1393 differentially expressed genes (651 upregulated, 742 downregulated). Upregulated genes were enriched in nucleolus- and nuclear pore-related cellular components and RNA-binding molecular functions, indicating enhanced ribosome biogenesis capacity. Downregulated genes were concentrated in DNA damage repair pathways and Mediator complex-mediated transcriptional regulation, suggesting alleviation of oxidative stress. Protein–protein interaction network analysis identified *mphosph10*, *utp11*, *nat10*, *emg1*, and *ftsj3* as core hub genes. Additionally, 44 differentially expressed transcription factors were identified, with Forkhead (forkhead.38), GATA (gata.4), and MYB (myb.40) significantly upregulated, indicating activation of a pro-developmental transcriptional program. These findings provide a theoretical basis for precision lipid nutrition formulation in *L. elongata* fish diets.

## Simple Summary

*Leptobotia elongata* (the elongate loach) is a rare freshwater fish native to China’s Yangtze River Basin that is listed as Vulnerable due to overfishing and habitat loss. A primary challenge for its commercial-scale cultivation is that captive females commonly exhibit developmental arrest of the ovary at stages I–II, with progression to stage III rarely achieved under artificial conditions, severely limiting the production of offspring. Dietary fats are essential for fish reproduction, providing the necessary components for ovarian follicle development and safeguarding reproductive cells from damage. In this study, captive elongate loaches were fed a diet enriched with fish oil, soybean phospholipid, and vitamin E for six months and it was found that this combination successfully promoted ovarian development from the developmentally arrested stages I–II to the actively growing stage III, whereas oocytes in the unsupplemented control group remained developmentally arrested. By analyzing differentially expressed genes in the ovaries, we discovered that this dietary intervention works through four coordinated biological pathways: boosting the cellular machinery needed to make proteins, reducing oxidative damage to reproductive cells, relieving a stress-related brake on gene activity, and switching on a network of genes that drive ovarian development. These findings provide practical guidance for formulating specialized diets to support the captive reproduction and conservation of this endangered species.

## 1. Introduction

*Leptobotia elongata* (Cypriniformes: Botiidae) is a subtropical freshwater benthic species endemic to China, with its distribution confined to the main stem and tributaries of the upper and middle Yangtze River [1]. *L. elongata* exhibits distinctive saddle-shaped markings along its lateral body surface and possesses a favorable muscle amino acid composition, contributing to its high nutritional quality. These combined traits underpin its dual economic value as a prized ornamental fish and a premium food fish, making it a highly regarded aquatic product [2]. However, due to the compounding effects of overfishing, habitat fragmentation, and hydraulic engineering construction, wild population resources have undergone continuous decline, and the species has been listed as Vulnerable (VU) on the China Species Red List [3]. Accordingly, the systematic development of artificial domestication and fully controlled artificial propagation of *L. elongata* represents a critical pathway for safeguarding its germplasm resources and achieving population recovery.

The quality of gonadal development in fish is the central determinant of artificial fish propagation, directly governing ovulation yield and fertilization rate, and thereby influencing the large-scale supply of fry. Nevertheless, *L. elongata* reared under artificial captivity commonly exhibits reproductive dysfunction manifested as retarded ovarian development or incomplete gonadal maturation [4], the nutritional etiology of which remains poorly understood. In fish reproductive physiology, lipids are the macronutrients with the highest energy density, providing not only energy substrates for rapid somatic growth and metabolic maintenance in fish, but also essential fatty acids, functional phospholipids, and cholesterol as bioactive components [5,6]. More critically, lipids play an indispensable regulatory role in reproduction: during vitellogenesis, lipids serve as material precursors for oocytes, stockpiling energy reserves and essential fatty acids for early embryonic development [7]. Simultaneously, sterols, particularly cholesterol, serve as direct precursors for synthesizing sex steroid hormones like estradiol. These hormones then drive ovarian development via cascade signaling along the hypothalamic–pituitary–gonadal (HPG) axis [8].

Evidence indicates that distinct classes of functional lipids, such as PUFA-rich fish oil, membrane-structuring phospholipids, and antioxidant vitamin E, exert both specific and synergistic regulatory functions during ovarian development in fish [9,10]. Despite this, the molecular mechanisms by which these exogenous lipids are metabolically transformed within *L. elongata* and subsequently regulate oocyte development through gene transcriptional networks remain virtually unexplored [11]. Existing research on *L. elongata* has predominantly focused on ecological behavior, morphological characteristics, and artificial propagation techniques [4,12]. However, systematic studies on the species-specific lipid requirements for fish gonadal development and the underlying molecular regulatory mechanisms are still lacking. This knowledge gap significantly impedes the rational development of specialized diets tailored for *L. elongata* fish and the precise restoration of its germplasm resources.

To address this, the present study integrated a systematic feeding trial (supplementing fish oil, phospholipid, and vitamin E) with ovarian transcriptomics in *L. elongata*. This approach aimed to compare their effects on ovarian development and elucidate the key genes as well as core signaling pathways involved in lipid nutritional regulation. This study aims to provide a theoretical basis for the precise lipid nutritional regulation of *L. elongata* fish while simultaneously offering a novel scientific perspective for elucidating the molecular mechanisms of reproductive metabolism in rare benthic fish species.

## 2. Materials and Methods

### 2.1. Ethics Statement

All animal experiments involving *L. elongata* conducted in this study complied with the standard operating procedures of the Institutional Animal Care and Use Committee (IACUC) of the relevant agricultural university and national research institution, and received formal ethical approval (HZAUFI-2022–0020). All efforts were made to minimize the suffering of experimental fish throughout the study. Prior to tissue collection, all *L. elongata* individuals were deeply anesthetized in a solution of 100 mg/L MS-222 (tricaine methanesulfonate, buffered to pH 7.0 with sodium bicarbonate), and subsequent procedures were performed on ice.

### 2.2. Experimental Fish

*L. elongata*, an endemic species of conservation concern from the upper reaches of the Yangtze River, was obtained from the Wudongde Fish Reproduction Base of China Three Gorges Corporation (Kunming, Yunnan, China), where the broodstock are maintained for artificial reproduction and population restoration. After 240 fish were transported to the laboratory, they were acclimated to the recirculating aquaculture system for two weeks and fed the control diet (CON) twice daily before the start of the feeding trial.

### 2.3. Experimental Diet Preparation

Isonitrogenous and isoenergetic compound basal diets were formulated for *L. elongata*. All dietary ingredients were ground and passed through a 60-mesh sieve (0.25 mm), thoroughly blended using a stepwise scaling-up mixing method, and cold-pressed into pellets (approximately 2 mm in diameter). Two experimental groups were established in this study (Table 1): the control group (CON) was fed the basal diet without any additional lipid supplementation; the mixed lipid group (MIX) received the basal diet supplemented with 6% fish oil, 6% soybean phospholipid, and 0.05% vitamin E. The prepared pellets were air-dried at low temperature (≤40 °C), vacuum-sealed, and stored at −20 °C in the dark until use. The proximate nutrient composition of each experimental diet, as determined by chemical analysis, is presented in Table 2.

### 2.4. Feeding Experiment

A total of 180 juvenile *L. elongata* with uniform size and good health (body weight: 6.75 ± 1.42 g; body length: 77.78 ± 5.94 mm) were randomly assigned to each experimental group and reared in an indoor recirculating aquaculture system (RAS) for a 6-month feeding trial (3 replicate tanks per group, 30 fish per tank). Fish were hand-fed to apparent satiation twice daily at 08:00 and 18:00 h; daily feed intake and uneaten feed residues were recorded throughout the trial. Water quality parameters were maintained as follows: water temperature 23–25 °C, dissolved oxygen ≥ 6.0 mg/L, pH 7.2 ± 0.3, and photoperiod 14L:10D. Throughout the 6-month feeding trial, fish in both groups exhibited normal feeding behavior, good health status, and no significant mortality (CON: 3.33%, 3/90; MIX: 2.22%, 2/90; *p*-value > 0.05).

### 2.5. Sample Collection

At the end of the feeding trial, experimental fish were subjected to a 24 h fasting period prior to sampling. Fish were anesthetized with 100 mg/L MS-222 (buffered to pH 7.0 with sodium bicarbonate), and the ovaries were subsequently dissected and removed. Mid-sections of ovarian tissue from each sample were fixed in 4% paraformaldehyde (pH 7.4) for hematoxylin and eosin (H&E) histological staining (*n* = 9 for each group: 3 healthy female fish with uniform body size were randomly selected from each of the 3 replicate tanks), while the remaining ovarian tissue was immediately snap-frozen in liquid nitrogen and stored at −80 °C for subsequent total RNA extraction (*n* = 9 for each group).

### 2.6. Histomorphological Observation

Fixed ovarian tissues were dehydrated through a graded ethanol series (70%, 80%, 90%, 95%, and 100%, 30 min each), cleared in xylene (2 × 15 min), infiltrated, and embedded in paraffin wax. Serial sections (5 μm thickness) were prepared and subjected to routine H&E staining, following a previously described protocol [13]. Sections were examined and photographed under a light microscope (Olympus BX53, Olympus, Tokyo, Japan), and the histomorphological characteristics of oocyte developmental stages were systematically documented.

### 2.7. Transcriptome Sequencing (RNA-Seq) and Bioinformatic Analysis

From each of the 3 replicate tanks, 3 healthy female fish with uniform body size were randomly selected. Approximately 20 mg of ovarian tissue was collected from each fish. Within the same tank, equal amounts of tissue from the 3 individuals were pooled to constitute a single biological replicate for transcriptomic sequencing. A total of 3 independent biological replicates were prepared per group (CON: C1, C2, C3; MIX: M1, M2, M3). Total RNA was extracted using the TRIzol reagent (Invitrogen, Carlsbad, CA, USA). RNA purity and concentration were assessed using a NanoDrop 2000 spectrophotometer (Thermo Scientific, Waltham, MA, USA) with OD_260_/_280_ ratios between 1.8 and 2.0 considered acceptable. RNA integrity was evaluated using an Agilent 2100 Bioanalyzer (Agilent, Santa Clara, CA, USA) with an RNA integrity number (RIN) ≥ 7.0 as the quality threshold. Qualified RNA samples were enriched for mRNA using oligo(dT) magnetic beads, and strand-specific sequencing libraries were constructed following the standard NEBNext Ultra II RNA Library Prep Kit protocol (NEB, Ipswich, MA, USA). Paired-end sequencing (150 bp) was performed on the Illumina NovaSeq 6000 platform (Illumina, San Diego, CA, USA).

Raw sequencing data were processed using Trimmomatic (v0.32) to remove adapters and filter low-quality reads (parameters: LEADING:3, TRAILING:3, SLIDINGWINDOW:4:15, MINLEN:36). The resulting clean reads were aligned to the *L. elongata* reference genome [11] using STAR (v2.7.8a). Gene expression levels were quantified using RSEM (v1.3.3) and normalized as transcripts per million (TPM). Differentially expressed genes (DEGs) were identified using the DESeq2 package (v1.46.0) with the following thresholds: |log_2_FoldChange| ≥ 1.5 and *p*-value < 0.05. Volcano plots were generated using the ggplot2 package (v4.0.1). Gene Ontology (GO) enrichment analysis of DEGs was performed using clusterProfiler (v4.18.4) (*p*-value < 0.05), and heatmaps of key gene expression profiles were generated using the pheatmap package (v1.0.12).

All annotated genes were ranked in descending order based on their log_2_(fold change) values derived from DESeq2 differential expression analysis. GSEA was conducted using the clusterProfiler (v4.18.4) package [14] in R (v4.3.2) with Gene Ontology (GO) gene sets, including biological process (BP), cellular component (CC), and molecular function (MF) ontologies. Phenotype-based permutations were performed to calculate the Normalized Enrichment Score (NES) and corresponding adjusted *p*-value. GO terms with |NES| > 1 and adjusted *p*-value < 0.05 were considered significantly enriched. The top significantly positively and negatively enriched GO terms were visualized using running enrichment score plots.

### 2.8. Protein–Protein Interaction Network Construction and Identification of Differentially Expressed Transcription Factors

Protein–Protein Interaction (PPI) Network Analysis: The protein sequences corresponding to the identified DEGs were submitted to the STRING database (https://string-db.org, version 11.5), with *Misgurnus anguillicaudatus* set as the reference species and a combined interaction score threshold of ≥0.7 applied to construct the DEG-based PPI network. The resulting interaction data were imported into Cytoscape (v3.10.0) for network visualization, in which node size was scaled proportionally to the degree value and node color was assigned according to the direction of gene regulation (upregulated: red; downregulated: blue; not significantly differentially expressed: grey). Topological parameters for each node, including degree and betweenness centrality, were calculated using the Network Analyzer plugin, and hub genes were defined based on degree ranking.

Identification of Differentially Expressed Transcription Factors (TFs): Transcription factor annotations for the *L. elongata* genome were retrieved from the AnimalTFDB database (https://string-db.org/, accessed on 25 April 2026, version 3.0). A systematic screening of the 1393 DEGs was performed by cross-referencing database annotations with domain-based nomenclature features to identify differentially expressed TFs with established TF family assignments. Identified TFs were classified according to their respective families, including zf-C2H2, MYB, Homeodomain, bHLH, bZIP, Forkhead, IRF, THAP, SAND, SWI/SNF, GATA, MBD, and STAT. A lollipop plot of the top 20 TFs ranked by the magnitude of differential expression was generated using ggplot2, with log_2_FoldChange on the x-axis and −log_10_(*p*-value) represented by dot size.

### 2.9. Quantitative Real-Time PCR (qRT-PCR) Validation

A total of 20 DEGs were selected to validate the RNA-Seq results by qRT-PCR. Total RNA was extracted as described in Section 2.7, and 500 ng of DNase-treated RNA per sample was reverse-transcribed into cDNA using the PrimeScript RT Reagent Kit with gDNA Eraser (TaKaRa, Shiga, Japan). Each qPCR reaction was performed in a total volume of 20 μL, comprising 10.0 μL TB Green Premix Ex Taq II (2×), 0.4 μL forward primer (10 μM), 0.4 μL reverse primer (10 μM), 2.0 μL cDNA template, and 7.2 μL ddH_2_O. The thermal cycling program consisted of an initial denaturation at 95 °C for 30 s, followed by 40 cycles of 95 °C for 10 s and 60 °C for 30 s, with a subsequent melting curve analysis to verify amplification specificity. All primers were designed using Primer Premier 6 software, and the sequences are listed in Table 3. Expression of β-actin was used as the reference gene for normalization. Relative gene expression levels were calculated using the 2^−ΔΔCt^ method, with the CON group set as the calibrator (expression = 1), and all other groups expressed as relative fold changes.

### 2.10. Blinding Procedure

A rigorous single-blind experimental design was implemented throughout the study to eliminate observer bias. Only the principal investigator (X.J.C. and M.G.) was aware of the group allocation throughout the entire experiment. Y.H.M., L.L., and J.G., who were responsible for daily feeding, water quality monitoring, and fish health management, were blinded to the group assignments; experimental diets were packaged in identical containers labeled only with neutral tank numbers with no indication of composition. Y.X.J., Y.H.M., and L.L., who performed the ovarian tissue dissection, histological sectioning, H&E staining, and oocyte developmental stage evaluation, were completely unaware of the treatment groups during all outcome assessment procedures. Y.X.J. and W.Q.C., who conducted transcriptomic data processing, bioinformatic analysis, and statistical testing, received only anonymized sample IDs with no corresponding group information until all primary analytical results were finalized.

### 2.11. Statistical Analysis

All quantitative data are presented as the mean ± standard deviation (Mean ± SD). Statistical analyses were performed using SPSS 26.0 (IBM, Armonk, NY, USA). The normality of the data distributions was assessed using the Shapiro–Wilk test, and homogeneity of variances was verified using Levene’s test. Comparisons between two groups were conducted using the Student’s *t*-test. Statistical significance was defined as *p*-value < 0.05, and high statistical significance as a *p*-value < 0.01.

## 3. Results

### 3.1. Histological Observation of Ovarian Tissue in L. elongata

**Figure 1:**
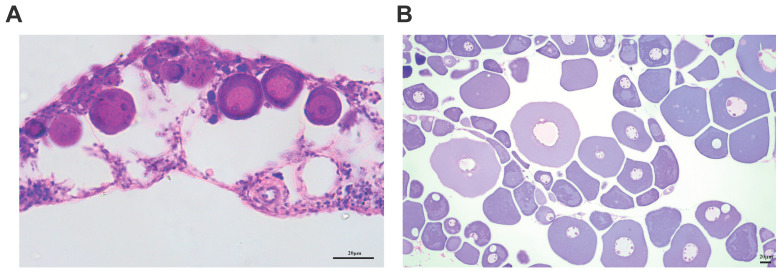
Histological sections of ovarian tissue from different experimental groups of *L. elongata*. (**A**) Control group (CON) consisting mainly of oocytes in phases I–II, with clearly visible nuclei; (**B**) mixed group (MIX) with oocytes entering phase III and a significantly larger cell size compared to the control group. Scale bar = 20 μm.

H&E staining revealed marked histomorphological differences in ovarian development among *L. elongata* subjected to different dietary lipid treatments following the 6-month feeding trial (Figure 1).

In the control group (CON), overall oocyte development was retarded, with the majority of oocytes arrested at stages I–II. Oocytes were comparatively small in diameter, with clearly visible nuclei in which the nucleoli were predominantly located within the nuclear interior (Figure 1A). In the mixed group (MIX), oocytes had advanced to stage III, exhibiting a marked increase in cell volume relative to the CON group. The nuclei were enlarged and nucleoli were clearly distinguishable, predominantly positioned at the center of the nucleus (Figure 1B). The proportion of stage III oocytes in the microscopic field was significantly elevated, with oocytes arranged in a regular pattern and no apparent signs of oocyte degeneration or atresia, indicating that ovarian development in the MIX group fish was substantially more advanced compared to the CON group.

### 3.2. Identification of Differentially Expressed Genes

**Figure 2:**
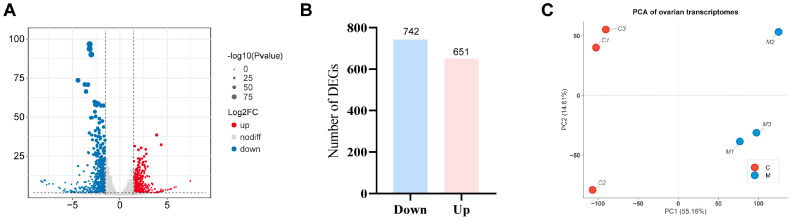
Differential expression analysis of ovarian transcriptomes between the CON and MIX groups in *L. elongata*. (**A**) Volcano plot of differentially expressed genes (DEGs). The x-axis represents log_2_fold change (log_2_FC) and the y-axis represents −log_10_ (*p*-value). Red dots indicate significantly upregulated genes (log_2_FC ≥ 1.5, *p*-value < 0.05), blue dots indicate significantly downregulated genes (log_2_FC ≤ −1.5, *p*-value < 0.05), and gray dots represent non-significantly differentially expressed genes. Dot size is proportional to −log_10_ (*p*-value). Horizontal and vertical dashed lines denote the significance and fold-change thresholds, respectively. (**B**) Bar chart showing the number of up- and downregulated DEGs. (**C**) Principal component analysis (PCA) score plot based on the whole-transcriptome expression profiles. PC1 and PC2 account for 55.16% and 14.61% of the total variance, respectively. Each group comprises three biological replicates (CON: C1–C3; MIX: M1–M3).

Principal component analysis (PCA) revealed distinct transcriptome-wide differences between the CON and MIX groups (Figure 2C). PC1 and PC2 accounted for 55.16% and 14.61% of the total variance, respectively. The two groups were completely separated along the PC1 axis, and the three biological replicates within each group clustered tightly together, indicating high sample preparation quality and statistical reliability of the between-group differences. Notably, sample C2 of the CON group exhibited a degree of dispersion along the PC2 axis, suggesting that a certain level of natural inter-individual variation in ovarian developmental status may exist within the control group.

Differential expression analysis was performed using DESeq2 with the following filtering criteria: |log_2_FoldChange| ≥ 1.5 and *p*-value < 0.05. A total of 1393 differentially expressed genes (DEGs) were identified (Figure 2A,B). Compared with the CON group, 651 genes were upregulated and 742 genes were downregulated in the MIX group. The slightly greater number of downregulated genes relative to upregulated genes suggests that mixed lipid supplementation exerted a broad and profound transcriptomic remodeling effect on the ovary at the global level.

### 3.3. Functional Enrichment Analysis of Differentially Expressed Genes

**Figure 3:**
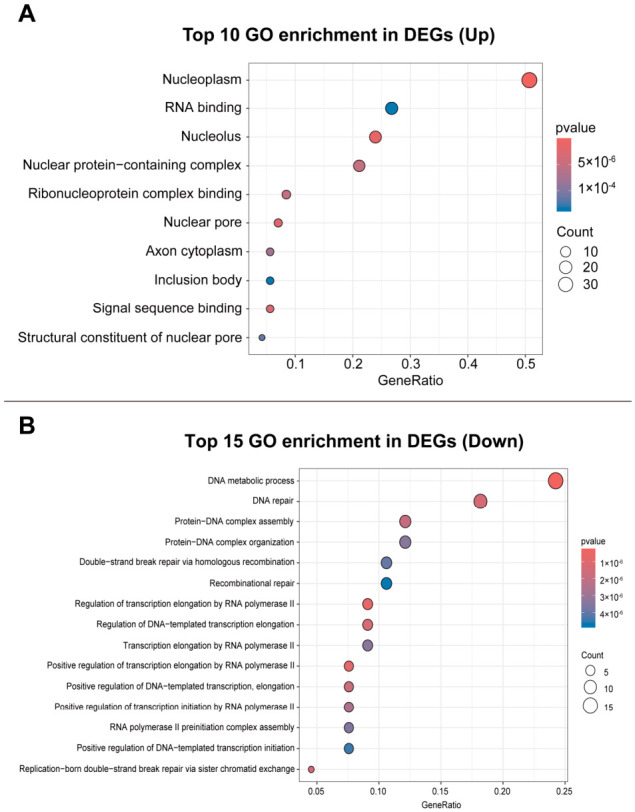
Gene Ontology (GO) enrichment analysis of differentially expressed genes (DEGs) in *L. elongata* ovary between the MIX and CON groups. (**A**) Dot plot of top 10 GO terms enriched in upregulated DEGs (MIX vs. CON). (**B**) Dot plot of the top 15 GO terms enriched in downregulated DEGs. The x-axis indicates GeneRatio, defined as the number of DEGs annotated to a given GO term divided by the total number of DEGs submitted for analysis. Dot size reflects the number of enriched genes (Count) and dot color represents the *p*-value (red: more significant; blue: less significant). GO terms with *p*-value < 0.05 are shown.

GO functional enrichment analysis was performed separately on the 651 upregulated DEGs and 742 downregulated DEGs; the results are presented in Figure 3.

Enrichment of upregulated DEGs. Significantly enriched terms were predominantly concentrated in two GO ontologies: cellular component (CC) and molecular function (MF). Within the CC ontology, nucleoplasm was the top-ranked term, with the highest number of annotated genes (*n* = 36); nucleolus, nuclear pore complex, and nuclear protein-containing complex also showed notable enrichment, collectively indicating an overall enhancement of nuclear architecture and function in the ovarian cells of the MIX group. Within the MF ontology, RNA binding, ribonucleoprotein complex binding, and signal sequence binding were significantly enriched, suggesting marked upregulation of molecular functions associated with RNA processing, transport, and protein targeting in the MIX group ovary. Taken together with the enrichment of nucleolus and nuclear pore terms in the CC ontology, these results indicate a global upregulation of intranuclear RNA metabolic activity and protein biosynthetic capacity in oocytes.

Enrichment of downregulated DEGs. Highly significant enrichment was detected across all three GO ontologies. Within the biological process (BP) ontology, DNA metabolic process and DNA repair were the terms with the highest number of annotated genes, with core genes including the classical DNA damage repair factors *rad51*, *xrcc1*, *ercc4*, *rad21*, and *cdc45*. Concurrently, regulation of transcription elongation by RNA polymerase II, double-strand break repair via homologous recombination, and recombinational repair were also significantly enriched, with the mediator complex subunits *med17*, *med20*, *med23*, *med24*, and *med28* identified as the central driver genes underlying these processes. Within the CC ontology, the core mediator complex and mediator complex exhibited exceptionally high fold-enrichment values, further corroborating the coordinated downregulation of MED family genes.

To further validate the over-representation analysis (ORA) results, we performed gene set enrichment analysis (GSEA). Specifically, GSEA revealed a significant positive enrichment of GO terms associated with nuclear architecture and RNA metabolism in the MIX group, including nuclear protein-containing complex, nucleolus, nucleoplasm, ribonucleoprotein complex binding, and RNA binding (Figure S1A). It confirmed that mixed lipid supplementation globally upregulated transcriptional programs related to ribosome biogenesis and protein synthesis, which are essential prerequisites for the rapid proliferative growth of stage III oocytes. Conversely, GO terms involved in cellular stress response and DNA damage repair, including cellular response to stress, DNA damage response, DNA metabolic process, and DNA repair, were significantly negatively enriched (Figure S1B). These results provide additional evidence that mixed lipid nutrition alleviates oxidative stress in ovarian cells.

### 3.4. Protein–Protein Interaction Network and Differentially Expressed Transcription Factor Analysis

**Figure 4:**
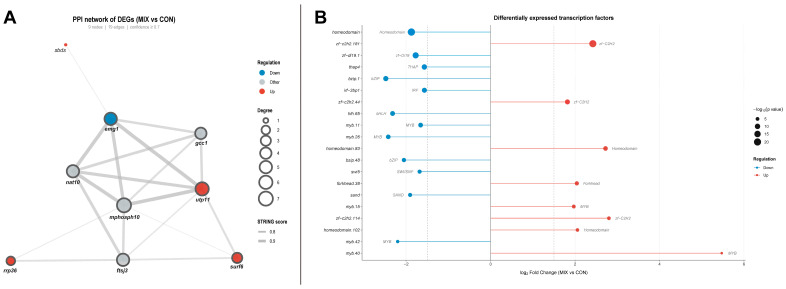
Protein–protein interaction (PPI) network and differentially expressed TFs of DEGs between the MIX and CON groups in *L. elongata* ovary. (**A**) PPI network of DEGs constructed using the STRING database (*Misgurnus anguillicaudatus* as reference, combined score ≥ 0.7). Node size represents degree (number of interactions) and node color indicates regulation direction (red: upregulated; blue: downregulated; gray: not significantly differentially expressed). Hub gene labels are shown in bold italic. (**B**) Lollipop plot of the top 20 most significantly differentially expressed TFs. The x-axis represents log_2_fold change (MIX vs. CON). Dot size is proportional to −log_10_ (*p*-value) and dot color indicates regulation direction. TF family annotations are shown in italic adjacent to each data point. Vertical dashed lines indicate the |log_2_FC| = 1.5 threshold.

The 1393 DEGs were submitted to the STRING database (confidence threshold ≥ 0.7) to construct a protein–protein interaction (PPI) network, yielding a core PPI network comprising 8 nodes and 19 interaction edges (Figure 4A). Topological analysis revealed that *mphosph10* (degree = 7) was the highest-degree hub gene within the network, followed by *utp11* (degree = 6), *emg1* (degree = 5), *ftsj3* (degree = 5), and *nat10* (degree = 5), along with *gcc1* (degree = 4), *surf6* (degree = 3), and *rrp36* (degree = 2).

With respect to the direction of regulation, the upregulated hub genes included *utp11*, *surf6*, and *rrp36*, while *emg1* was identified as a downregulated hub gene. Notably, the functional annotations of these hub genes were highly convergent: UTP11, EMG1, FTSJ3, NAT10, MPHOSPH10, and SURF6 are all nucleolar proteins involved in ribosomal RNA (rRNA) processing, 18S rRNA methylation, and ribosomal small and large subunit assembly, while *rrp36* encodes a component of the rRNA processing complex. Collectively, the functional annotations of these hub genes converged on ribosome biogenesis, consistent with the GO enrichment results of upregulated DEGs.

Based on domain-based nomenclature features of gene names, a total of 44 differentially expressed transcription factors (TFs) were identified among the 1393 DEGs, spanning 13 TF families including zf-C2H2, MYB, Homeodomain, bHLH, bZIP, Forkhead, IRF, THAP, SAND, SWI/SNF, GATA, MBD, and STAT (Figure 4B).

Among the 22 upregulated TFs, zf-c2h2.181 was the most statistically significant differentially expressed TF, belonging to the zinc finger C2H2 family, members of which are broadly implicated in transcriptional activation and chromatin remodeling. Homeodomain.83 and homeodomain.102 were significantly upregulated; homeodomain TFs are known to play critical roles in establishing the developmental axis of oocytes. Forkhead.38 was also significantly upregulated; members of the Forkhead family (FOXO subfamily) have been demonstrated to regulate follicular survival and ovarian growth via the PI3K–Akt signaling axis. Although myb.40 (log_2_FC = 5.47) showed relatively lower statistical significance, it exhibited the highest fold-change among all identified TFs, suggesting that this MYB family member may exert a specific transcriptional activation function in the ovaries from the MIX group. Additionally, the upregulation of gata.4 implies the involvement of GATA transcription factors in the regulation of ovarian cell fate.

Among the 22 downregulated TFs, homeodomain was the most statistically significant downregulated TF. Multiple MYB family members (myb.11, myb.26, myb.42), bzip.1 and bzip.48 (bZIP family), irf-2bp1 (IRF family), swi5 (SWI/SNF chromatin remodeling complex), and hlh.65 (bHLH family) were all significantly downregulated in the MIX group.

### 3.5. Identification of Genes Associated with Ovarian Development in L. elongata

**Figure 5:**
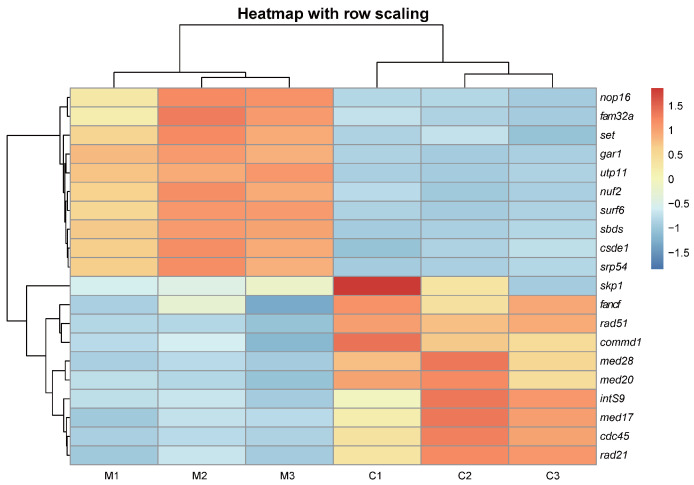
Hierarchical clustering heatmap of differentially expressed genes in the ovaries of *L. elongata* between the control and experimental groups. The control group was fed a commercial diet, in which ovarian development remained predominantly at stages I–II, while the experimental group was fed the same commercial diet supplemented with fish oil, soybean phospholipid, and vitamin E, in which ovarian development advanced to stage III. The heatmap displays the expression profiles of 20 selected differentially expressed genes (10 upregulated and 10 downregulated) across the control group (C1, C2, C3) and the experimental group (M1, M2, M3). Each row represents a gene and each column represents a sample. The color scale ranges from blue (low relative expression) to red (high relative expression) based on row-scaled Z-scores (scale: −1.5 to 1.5). Both rows and columns are arranged by hierarchical clustering, with dendrograms indicating the clustering relationships among genes and samples.

To validate the reliability of the transcriptomic differential expression analysis and to visualize the expression patterns of candidate genes in stage I–II (CON) versus stage III (MIX) ovaries of *L. elongata*, hierarchical clustering heatmap analysis was performed on 20 selected DEGs (10 upregulated and 10 downregulated) (Figure 5). The results demonstrated that all samples were clearly segregated into two distinct clusters based on their gene expression profiles, with the control group and the experimental group each forming a discrete cluster, indicating robust between-group consistency and within-group reproducibility of the differential expression patterns.

Among the upregulated genes, *nop16*, *fam32a*, *skp1*, *gar1*, *utp11*, *nuf2*, *surf6*, *sbds*, *csde1*, and *srp54* all exhibited relatively high expression levels in the stage III group (MIX) (represented in red in the heatmap) and comparatively low expression levels in the stage I–II group (CON) (represented in blue). Conversely, among the downregulated genes, *set*, *fancf*, *rad51*, *commd1*, *med28*, *med20*, *ints9*, *med17*, *cdc45*, and *rad21* displayed the opposite expression trend, being highly expressed in the stage I–II group (CON), and lowly expressed in the stage III group (MIX). These expression patterns were in strong concordance with the results of the transcriptomic differential expression analysis, further suggesting that these genes may be associated with ovarian development in *L. elongata*.

**Figure 6:**
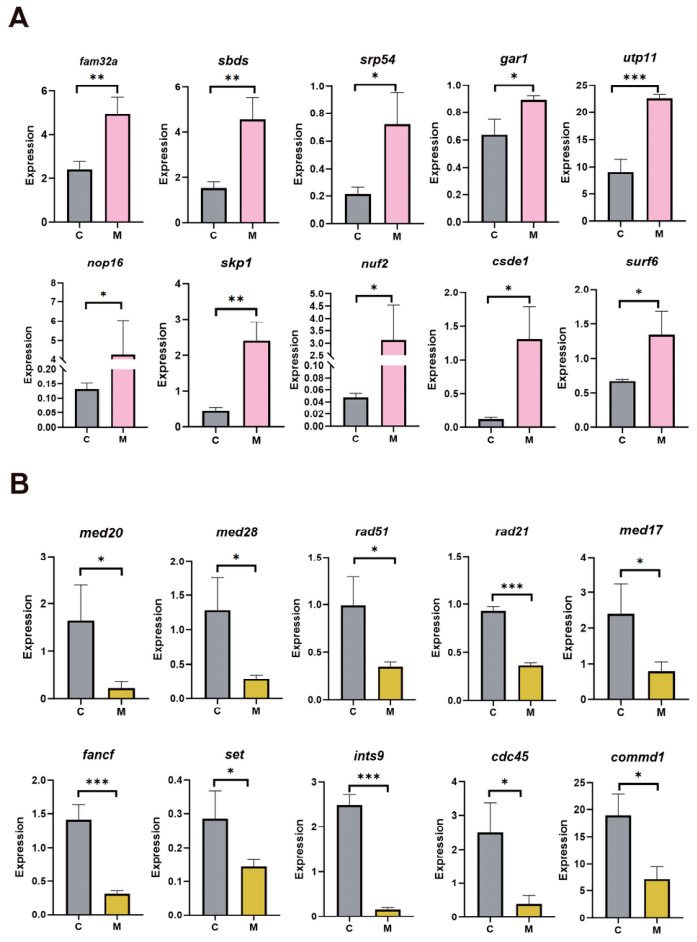
Validation of differentially expressed genes in the ovaries of *L. elongata* between the control and experimental groups by quantitative real-time PCR (qRT-PCR). The control group (C) was fed a commercial diet, where ovarian development remained predominantly at stages I–II, while the experimental group (M) was fed the same commercial diet supplemented with fish oil, soybean phospholipid, and vitamin E, where ovarian development advanced to stage III. (**A**) Relative expression levels of 10 upregulated differentially expressed genes (pink bars); (**B**) relative expression levels of 10 downregulated differentially expressed genes (yellow bars). The control group is shown in gray bars. Data are presented as mean ± SD, *n* = 3. * *p*-value < 0.05, ** *p*-value < 0.01, *** *p*-value < 0.001.

To further validate the accuracy of the transcriptomic differential expression analysis and to elucidate the regulatory effects of dietary lipid supplementation on the expression of ovarian development-related genes in *L. elongata*, qRT-PCR was performed on the 20 selected DEGs (Figure 6). The quantification results for the 10 upregulated genes were in strong concordance with the transcriptomic data (Figure 6A): compared with the CON group, the expression levels of *fam32a*, *sbds*, *srp54*, *gar1*, *utp11*, *nop16*, *skp1*, *nuf2*, *csde1*, and *surf6* were all significantly elevated in the ovaries of the MIX group (*p*-value < 0.05), with utp11 showing the most pronounced difference (*p*-value < 0.001). These findings indicate that dietary supplementation with fish oil, soybean phospholipid, and vitamin E significantly promoted the transcriptional expression of these genes. The majority of these genes are involved in biological processes including ribosome biogenesis, RNA binding, and nucleocytoplasmic transport, consistent with the GO enrichment results of upregulated DEGs (Section 3.3). Among the 10 downregulated genes (Figure 6B), the expression levels of *med20*, *med28*, *rad51*, *rad21*, *med17*, *fancf*, *set*, *ints9*, *cdc45*, and *commd1* were all significantly reduced in the ovaries of the MIX group relative to the CON group (*p*-value < 0.05), with *rad21* and *fancf* exhibiting the most marked differences (*p*-value < 0.001), and *ints9* also reaching a highly significant level (*p*-value < 0.001). These genes are primarily enriched in pathways associated with DNA damage repair, transcriptional regulation, and cell cycle control, consistent with the GO enrichment results of downregulated DEGs (Section 3.3). Taken together, the qRT-PCR results were in strong agreement with the transcriptomic analysis, further confirming that mixed lipid supplementation significantly modulated the expression levels of genes involved in DNA damage repair and transcriptional regulation in the ovaries of *L. elongata*.

## 4. Discussion

### 4.1. Mixed Lipid Nutrition Promotes Proliferative Oocyte Growth and Ovarian Development in L. elongata

Proliferative oocyte growth represents an obligatory prerequisite phase for the initiation of vitellogenesis during fish ovarian development in teleosts. Vitellogenesis is fundamentally defined as the process by which vitellogenin (Vtg), synthesized in the liver, is transported via the bloodstream to the ovary, where it is internalized by oocytes through receptor-mediated endocytosis and subsequently processed and stored [15]. Ovarian development imposes stringent demands on lipid supply. Specifically, phospholipids serve as the structural scaffold of yolk lipoprotein granules [16]; n-3 PUFAs (especially EPA and DHA) act as essential fatty acid reserves for early embryos; and vitamin E protects these PUFA-rich phospholipids from oxidative degradation by scavenging ROS [9,10].

Histological analysis revealed that oocytes in the MIX group had advanced to stage III from stages I–II. These oocytes displayed increased volume and distinct nucleoli, but no cytoplasmic yolk granule deposition, indicating the completion of proliferative growth and entry into the pre-vitellogenic phase (Figure 1). In contrast, oocytes in the control (CON) group remained at earlier developmental stages. This gonad-promoting effect is consistent with previous reports in yellow catfish (*Pelteobagrus fulvidraco*), where dietary nutritional composition significantly altered the plasma Vtg levels and the gonadosomatic index (GSI) [17]. In cyprinid fishes, LH signaling plays a predominant role in both vitellogenesis and ovarian development [18]. n-3 PUFAs derived from fish oil promote sex steroid hormone synthesis by maintaining membrane fluidity and ensuring adequate cholesterol availability, thereby driving hepatic Vtg synthesis and secretion [8]. Hepatic transcriptomic analysis in pond loach (*Misgurnus anguillicaudatus*) further demonstrated that dietary fish oil quality directly influences the expression of hepatic lipid metabolism-related genes (including *fatp*, *fabp*, and *pparg*) [19], suggesting that high-quality fish oil in *L. elongata* may activate PPARβ-mediated lipid-sensing pathways [20], thereby upregulating the transcriptional programs associated with Vtg synthesis. Furthermore, IGFBP1a-mediated nutritional deficiency signaling can suppress the expression of GnRH3, FSH, and LH along the hypothalamic–pituitary–gonadal (HPG) axis [21], indicating that insufficient functional lipid supply may be a contributing factor to the retarded ovarian development observed in the CON group. The lipid intervention in the MIX group likely helped restore this metabolic imbalance, thereby reactivating gonadotropic signaling along the HPG axis. In addition, fatty acid β-oxidation, as a critical energy metabolic pathway in oocytes, has also been implicated in leptin-mediated regulation of ovarian development [22].

Collectively, these findings suggest that the mixed lipid formulation synergistically promotes the proliferative developmental progression of oocytes in *L. elongata* through multiple regulatory tiers, facilitating the transition of gonads from the developmentally arrested stages I–II to the actively growing stage III state. These results provide experimental evidence supporting precision lipid formulation strategies in fish diets.

### 4.2. Upregulation of Ribosome Biogenesis as the Core Molecular Basis for Mixed Lipid-Promoted Oocyte Development

Ribosome biogenesis is the most energetically demanding anabolic process within the cell. It is centered in the nucleolus and encompasses a series of highly coordinated molecular events, including rDNA transcription, pre-rRNA processing, ribosomal protein assembly, and the nucleocytoplasmic export of mature ribosomal subunits [11]. Within this process, THO complex-mediated RNA nucleocytoplasmic export is critical for the cytoplasmic translocation of mature ribosomal subunits, and its components exhibit developmental stage-dependent expression patterns in the carp ovary [23].

In the present study, the hub genes identified from the protein–protein interaction (PPI) network—*utp11*, *emg1*, *nat10*, *ftsj3*, *mphosph10*, *surf6*, and *rrp36*—were functionally concentrated in ribosome biogenesis, representing one of the most significant core findings of this study. Ribosome biogenesis holds considerable strategic importance during oocyte development in teleosts: during the proliferative growth phase preceding vitellogenesis, oocytes must substantially expand their ribosome biogenesis capacity; subsequently, during vitellogenesis itself, oocytes are required to synthesize large quantities of yolk proteins, oocyte-specific RNA-binding proteins, and maternal regulatory factors, placing exceptionally high quantitative demands on the translational machinery [24]. Transcriptomic studies of the ovary in siluriform fishes have demonstrated that ovary-preferentially expressed genes are highly enriched in pathways related to RNA transport and ribosome biogenesis [25]. Positive selection analysis of the ovarian transcriptome in European anchovy (*Engraulis encrasicolus*) further revealed that RNA methylation modification and ribosome biogenesis represent the most significantly enriched functional categories among positively selected ovarian genes [26]. In the pond loach (*Misgurnus anguillicaudatus*), comparative transcriptomic analysis between diploid and tetraploid ovaries similarly highlighted the critical roles of ribosome biogenesis and RNA processing pathways during oocyte development [27].

At the molecular function level, NAT10 catalyzes ac4C modification of 18S rRNA, which is indispensable for the correct assembly of the small ribosomal subunit and translational fidelity; UTP11 participates in SSU processome assembly; and EMG1 (NEP1) functions as a pseudouridine synthase at a critical position within 18S rRNA, with loss-of-function mutations causing ribosomopathy. In rainbow trout (*Oncorhynchus mykiss*), the ribosomal protein RpL10a is specifically and highly expressed in chromatin–nucleolus stage oocytes and is sensitive to estradiol treatment [28], suggesting that the regulation of ribosomal protein genes during ovarian development may represent a conserved mechanism in teleosts. In Nile tilapia, genome-wide cytoplasmic ribosomal protein genes similarly display gonad-biased expression patterns, further supporting this conservation [29]. In zebrafish, Rbpms2 promotes nucleolar amplification via the mTORC1–Gator2 pathway to drive ribosome biogenesis and ovarian fate specification [30]. Notably, this finding offers strong cross-species support for our observation that upregulated genes in *L. elongata* were enriched in Gene Ontology (GO) terms related to the nucleolus and nuclear pore.

Taken together, these findings suggest that mixed lipid treatment substantially enhanced ribosome biogenesis capacity in stage III oocytes of *L. elongata*, thereby establishing the translational machinery required for the large-scale protein synthesis that underpins both proliferative oocyte growth and the subsequent transition toward vitellogenesis.

### 4.3. Mixed Lipid Nutrition Downregulates DNA Damage Repair and Mediator-Associated Stress Transcription Pathways by Alleviating Oxidative Stress

GO enrichment analysis of downregulated differentially expressed genes (DEGs) in the present study revealed a functionally convergent pattern: DNA metabolic processes, homologous recombination repair, and Mediator complex-associated transcriptional regulation were persistently enriched biological processes in the CON group ovary. The core driver genes included classical DNA damage response (DDR) factors such as *rad51*, *fancf*, *rad21*, *cdc45*, and *xrcc1*, as well as Mediator subunits including *med17*, *med20*, *med23*, *med24*, and *med28* (Figure 3). qRT-PCR validation further confirmed the significant downregulation of these genes in the MIX group (Figure 6).

Collectively, these enrichment patterns suggest that under control dietary conditions (CON group), ovarian cells were subjected to sustained DNA damage stress, with the DNA repair pathway highly activated and the transcriptional regulatory machinery in a stress-induced upregulated state. In contrast, mixed lipid supplementation (MIX group) likely alleviated oxidative damage stress in ovarian cells by providing adequate antioxidant protection (vitamin E) and membrane lipid homeostasis (soybean phospholipid and fish oil), thereby enabling the downregulation of stress-associated genes and facilitating the reallocation of cellular resources toward the anabolic processes required for normal oocyte development.

Interpreting this pattern requires the consideration of the biological context of meiosis and DNA damage responses in teleosts. During normal meiotic progression in fish, Rad21 (a cohesin subunit) ensures correct homologous chromosome pairing, Cdc45 participates in DNA replication initiation, and Fancf operates within the Fanconi anemia (FA) pathway to mediate DNA interstrand crosslink repair. Studies on temperature-induced sex reversal in zebrafish demonstrated that aberrant activation of FA pathway genes is closely associated with the disruption of gonadal differentiation [31]. In cyprinid polyploid fish, incomplete double-strand break repair has been identified as a direct cause of oocyte meiotic arrest and apoptosis [32].

Against this background, the sustained hyperactivation of DNA repair pathways in the CON group ovary may be driven by “nutritional oxidative stress”: under basal dietary conditions, PUFA-rich phospholipids in oocyte membranes lack antioxidant protection from vitamin E and are therefore susceptible to ROS attack, with the resultant lipid peroxidation products (e.g., 4-HNE and MDA) forming DNA adducts that activate the DDR. This is consistent with the mechanism reported in pond loach, in which H_2_O_2_ stress activates the apoptotic pathway via the CHOP–ATF4–BAX axis [33]. In the MIX group, vitamin E interrupts the lipid peroxidation chain reaction by scavenging free radicals, while soybean phospholipid and fish oil maintain membrane structural homeostasis by providing high-quality phospholipids. The synergistic action of these three components reduces the overall level of oxidative damage, enabling the DDR program to be “disarmed” and allowing cellular resources to be redirected toward anabolic programs.

The coordinated upregulation of Mediator complex subunits in the CON group similarly reflects a stress-driven transcriptional state. As a general coactivator of RNA polymerase II, the Mediator complex exerts precise temporal control over transcription during fish development. Studies on zebrafish *med12* mutants have demonstrated that loss of Mediator subunits results in aberrant timing and amplitude of target gene transcription [34]. The coordinated upregulation of MED subunits in the CON group likely represents the maintenance of a stress-responsive transcriptional network under sustained oxidative stress; their global downregulation in the MIX group signals a programmatic switch from “stress maintenance” to “developmental progression”.

### 4.4. Differentially Expressed Transcription Factors Reveal a Lipid Nutrition-Driven Switch in the Ovarian Transcriptional Program

A total of 44 differentially expressed transcription factors (TFs) spanning 13 families were identified in the present study. Upregulated TFs were represented primarily by the Forkhead (forkhead.38), GATA (gata.4), and MYB (myb.40) families, while downregulated TFs were dominated by Homeodomain family members, multiple MYB members (myb.11, myb.26, myb.42), and the bHLH family (hlh.65). This overall landscape is consistent with a transcriptional program switch from developmentally arrested stage I–II oocytes to the actively proliferating stage III state.

Among the upregulated TFs, the marked upregulation of forkhead.38 is of particular biological significance. Members of the FOXL2 and FOXO subfamilies within the Forkhead family are central transcription factors for ovarian fate specification in teleosts. In zebrafish, Foxl2l drives the differentiation of germline progenitor cells toward the oocyte lineage, thereby determining female fate [35]. In yellow catfish, FOXL2 directly drives ovarian estrogen synthesis by activating the promoter of *cyp19a1a* (the ovarian aromatase gene) [36]. In pond loach, *cyp19a1a* expression peaks during vitellogenesis under fine-scale regulation by DNA methylation [37]. This aligns with our inference that the upregulation of Forkhead-family transcription factors promotes the initiation of estrogen synthesis during the oocyte proliferative growth phase. In large-scale loach (*Paramisgurnus dabryanus*), knockdown of *foxl2a* directly induces female-to-male sex reversal [38], further underscoring the conserved role of Forkhead-family TFs in maintaining ovarian fate in cypriniform fishes.

The upregulation of gata.4 is consistent with the established functions of the GATA family in teleost ovarian development. In yellow catfish, GATA transcription factors serve as regulators of the *tspo* and *smad4* gene promoters, participating in the transcriptional control of ovarian steroidogenesis [36]. Additionally, the nuclear receptor transcription factor DAX1 regulates the expression of steroidogenesis-related genes during ovarian development in pond loach and is an important factor in maintaining ovarian function [39]. The pronounced upregulation of myb.40 (log_2_FC = 5.47) warrants attention; MYB family transcription factors are broadly involved in cell proliferation and differentiation programs, and their specific functions during oocyte vitellogenesis in teleosts remain to be further explored.

Among the downregulated TFs, the downregulation of hlh.65 (bHLH family) is relevant to the functional significance of FIGLA (Factor In the Germ Line Alpha). In Nile tilapia, FIGLA maintains the ovarian differentiation trajectory by antagonizing spermatogenesis-associated genes [40]. Its elevated expression in the CON group may reflect a compensatory pro-developmental signal in the context of impaired ovarian development, which is subsequently resolved upon the restoration of normal ovarian development in the MIX group. The downregulation of multiple Homeodomain-family TFs further suggests the presence of aberrantly activated gonadal stress signaling in the CON group ovary. ATAC-Seq analysis of cyprinid ovaries showed that chromatin accessibility at FOXL2 and SF1 binding sites is specifically enriched and correlates positively with the expression of ovarian fate genes like *cyp19a1a* [41]. Thus, this epigenetic-level evidence supports our mechanistic inference that the upregulation of Forkhead- and GATA-family TFs drives ovarian development in the present study.

These findings suggest that a stress-responsive and transcriptionally repressive TF network was maintained in a persistently active state in the CON group ovary. Mixed lipid supplementation appears to suppress these stress-associated transcriptional programs, thereby redirecting cellular transcriptional resources toward the pro-developmental TF network required for normal oocyte progression.

It should also be acknowledged that although the present study demonstrated a gonad-promoting effect of the mixed lipid supplementation, the regulatory efficacy of dietary lipids in fish reproduction reflects an intricate balance among individual fatty acid (FA) groups, and in particular, among the ratios of docosahexaenoic acid (DHA), eicosapentaenoic acid (EPA), and arachidonic acid (AA), which collectively modulate steroidogenic hormone synthesis, prostaglandin signaling, and oocyte membrane biophysical properties. The natural lipid composition adopted here was not pre-tailored to a species-specific reference FA profile, which represents a shortcoming of the present design. To enable a truly precision diet formulation for *L. elongata* broodstock, future studies should perform comprehensive FA analyses of wild-living individuals sampled across successive stages of ovulation and spermatogenesis, and use these in vivo reference profiles as the compositional benchmark against which broodstock diets are formulated.

Taken together, the present study systematically dissected, at the level of the ovarian transcriptome, the molecular mechanisms by which mixed lipid nutrition (fish oil + soybean phospholipid + vitamin E) promotes ovarian development in *L. elongata,* revealing four synergistic regulatory axes: upregulation of ribosome biogenesis, downregulation of DNA damage repair pathways, disarmament of Mediator-associated stress transcription programs, and activation of a pro-developmental transcription factor network. This multi-tiered molecular regulatory landscape provides a theoretical basis for precision lipid nutrition formulation in *L. elongata* fish compound diets, and offers a new scientific perspective for deciphering the molecular mechanisms underlying the reproductive metabolism of rare benthic fish species. Future studies may further employ gene function intervention approaches (e.g., RNAi or CRISPR) to verify the causal roles of hub genes (*utp11*, *nat10*, etc.) in *L. elongata* oocyte development and integrate lipidomics and proteomics to elucidate the upstream molecular mechanisms by which lipid nutritional signals activate the ribosome biogenesis pathway.

## 5. Conclusions

In summary, dietary supplementation with fish oil, soybean phospholipid, and vitamin E significantly promoted ovarian development in *L. elongata*, advancing oocytes from the arrested stages I–II to the proliferative growth stage III. Transcriptomic analysis revealed four synergistic molecular mechanisms underlying this transition: upregulation of ribosome biogenesis, downregulation of DNA damage repair pathways, relief of Mediator complex-mediated transcriptional suppression, and activation of a pro-developmental transcription factor network centered on Forkhead, GATA, and MYB family members. These findings provide a theoretical basis for precision lipid nutrition formulation in *L. elongata* fish diets and offer a novel perspective for understanding the reproductive metabolism of rare benthic fish species.

## Figures and Tables

**Table 1:** Diet formulation.

	CON	MIX
Fish meal	50	50
Shrimp meal	8	8
Squid meal	20	20
Multivitamins and minerals	3	3
Soybean oil	6	0
Fish oil	0	6
Phospholipid oil	0	6
Vitamin E	0	0.05
Choline chloride	1	1
Starch	2	2
Calcium dihydrogen phosphate	2	2
Dextrin	8	1.95

Values are expressed as percentage (%) of the diet on an as-fed basis. CON, control diet in which 6% soybean oil was used as the lipid source; MIX, experimental diet in which soybean oil was replaced by a mixture of fish oil (6%) and phospholipid oil (6%), with 0.05% vitamin E supplemented as an antioxidant, and the level of dextrin adjusted accordingly to keep the diets isonitrogenous and approximately isoenergetic.

**Table 2:** Proximate composition of the diet (% of dry matter).

Ingredient	CON	MIX
Dry matter	91.6	91.6
Crude protein	51.2	50.7
Crude lipid	11.2	15.7
Ash	6.7	6.4

Values are presented as means of triplicate analyses. CON and MIX are defined as in Table 1. Dry matter, the residual content after the removal of moisture; crude protein, total nitrogenous compounds calculated as N × 6.25; crude lipid, total fat content including triglycerides, phospholipids and other lipid-soluble components; ash, total inorganic mineral residue.

**Table 3:** Primer sequences validated by qRT-PCR.

Primer	Sequence (5′ to 3′)
β-actin-F	GTCGTGACCTGACAGACTACCT
β-actin-R	GGATGAAGAGGAGGCAGCAGTA
down-CDC45-F135	GGAGTGTTCAGTCTTGATGGTT
down-CDC45-R135	AATTGTGTACTTGCCAGGAGAC
down-MED28-F113	TGTGTCCTGAATTGGTGAGATC
down-MED28-R113	TGCTTCCGTGAAACCTCCT
down-RAD51-F114	TGTGTGTCCTGAATTGGTGAGA
down-RAD51-R114	GCTTCCGTGAAACCTCCTTCC
down-RAD21-F107	CCATCCTGACCTTGAGCAGTCT
down-RAD21-R107	GCCCACCAAGAAGCTGATGATG
down-MED20-F80	GACCTCCACTGAAATCCCTCTA
down-MED20-R80	GCTACCGTTACTGCGACTTC
down-COMMD1-F96	GTGAGAAACGCTTCGAGTTG
down-COMMD1-R96	AGGAAGCACGACCACCAT
down-MED17-F127	CCCAACTTCCGTTTGCCCTTT
down-MED17-R127	CGCTATTCCGACCAATTCACCA
down-FANCF-F149	CAGCATAGCCGCAGCACAAG
down-FANCF-R149	CAGCCTCAGCCTCACACTCTT
down-SET-F144	TCCACTGCATGTTCCTGCTTCA
down-SET-R144	CGCTGATAACCGCCGATGAGT
down-INTS9-F86	TGTTCCGTCCGCAGATACC
down-INTS9-R86	AGGGCACTACCATAACCATCC
UP-NOP16-F138	ACAGCACATGGTGAGGGAACA
UP-NOP16-R138	TGCCATTGCCTGAGGTGACA
UP-SKP1-F118	CAACAGCACCAGACTCCTTCAC
UP-SKP1-R118	AATCCGCTGGCATCATTGTCC
UP-NUF2-F112	GGCTCAACGGCACCAGGTAT
UP-NUF2-R112	GCACACGCTCAACTTGTCACA
UP-CSDE1-F145	TCTCCTTCACCACATCTCCTCT
UP-CSDE1-R145	CCGCCTACTCGTCTGTCAATC
UP-FAM32A-F80	CAGGCACTACGGCTGTCTT
UP-FAM32A-R80	CATTGGCGGTTCCTTGTTGA
UP-SURF6-F81	CTTCTCTACCACTTGCTGACTC
UP-SURF6-R81	GCATCACTGAAGAGGAAGGAG
UP-SBDS-F99	CTGCCTCCATCCAGTGAAGA
UP-SBDS-R99	CACAAGCCAACGCACATCA
UP-SRP54-F93	GACCCTTCTTCTTGCCCTTCT
UP-SRP54-R93	AACGCAGCCACTCATTCCT
UP-GAR1-F101	CAGAATGAACTGCCTCAACCT
UP-GAR1-R101	CAAGCGGTGCGTAAGTGT
UP-UTP11-F96	ACTGCTTGACACCGCTCTG
UP-UTP11-R96	GGCACAATCGCTGGTATTCTC

## Data Availability

The original contributions presented in this study are included in the article. Further inquiries can be directed to the corresponding authors.

## Supplementary Materials

The following supporting information can be downloaded at: https://www.mdpi.com/article/10.3390/ani16111604/s1, Figure S1: Gene Set Enrichment Analysis (GSEA) of differentially expressed genes (DEGs) between MIX and CON groups. (A) Score plot of top 5 GO terms enriched in upregulated DEGs (MIX vs. CON). (B) Score plot of top 7 GO terms enriched in downregulated DEGs. The x-axis represents the rank of all genes ordered by log_2_(fold change) (MIX vs. CON), and the y-axis represents the running enrichment score.
