# Supplementary material for: Transcriptomic Mechanisms Underlying Dietary Fish Oil, Phospholipid, and Vitamin E Supplementation in Promoting Ovarian Development in Leptobotia elongata
Source: Animals (Basel). 2026 May 25;16(11):1604. doi: 10.3390/ani16111604 (PMC13255876; doi:10.3390/ani16111604)
Supplement: Supplementary file 1 [file animals-16-01604-s001.zip › animals-4298628-supplementary.pdf]

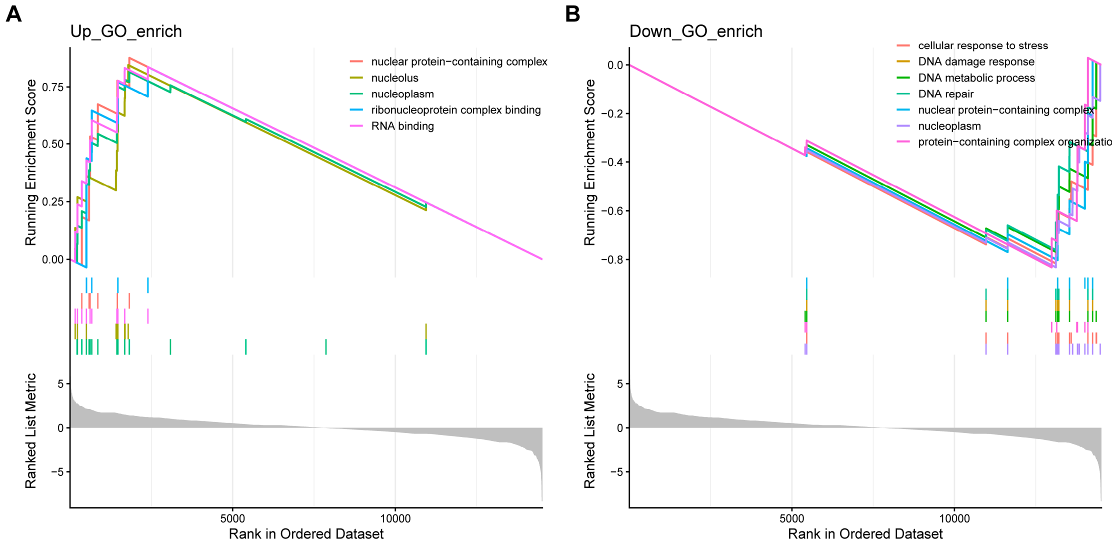

Figure S1. Gene Set Enrichment Analysis (GSEA) of differentially expressed genes (DEGs) between MIX and CON groups.

(A) Score plot of top 5 GO terms enriched in upregulated DEGs (MIX vs. CON). (B) Score plot of top 7 GO terms enriched in downregulated DEGs. The x-axis represents the rank of all genes ordered by $\log_2$(fold change) (MIX vs. CON), and the y-axis represents the running enrichment score.
